# The Cinematograph at Charing Cross Hospital

**Published:** 1913-01-04

**Authors:** 


### THE CINEMATOGRAPH AT CHARING CROSS HOSPITAL.

This year the Christmas festivities at Charing Cross Hospital followed in the main the plan which had proved so successful in previous years. Two factors, however, tended to reduce the number of visitors to the wards in the afternoon of Christmas Day; one was the wretched weather, the other was the limitation of the number of the patients' friends to two only. The earliest item on the programme was the singing of carols by the Choir of St. Martin-in-the-Fields. The special dainties associated with the day were, of course, doled out without stint to all those able to partake of these complex proteids. In the afternoon the wards were invaded by numerous friends both of the staff and the patients. Entertainers were almost as plentiful as lookers-on, and the Christmas spirit was very much in evidence. Many excellent performances were given over and over again, and the performers, even at "closing time," showed no diminution in their unselfish efforts. Among the parties going round was a group from the Garrick Theatre, who seemed to be in high favour. The whole of the arrangements were very creditable to those responsible, and especially to the Resident Medical Officer, Mr. Bertram Lloyd, who, although quite a newcomer and previously unconnected with Charing Cross Hospital, showed that he was not going to be in any way behind his predecessors in helping everyone to enjoy themselves. The wards were all tastefully and some elaborately decorated, but the Golding Ward had a specially pleasing appearance. This ward is one of the old wards which has lately been repainted in pure white, to the great advantage of its inmates.

Charing Cross Hospital is one of the institutions which received the gift of a pathescope from the well-known firm of cinematograph film manufacturers. This machine, which is exceedingly compact and portable, proved a great boon. With very little preparation anyone can give an effective moving-picture show in any room or ward. As the machine generates its own illumination, users are independent of dangerous and cumbersome sources of light. Its portability ensures that every ward shall have its own show, and the resident medical officers have proved themselves willing operators. The apparatus has its home in the board-room, from which it is removed whenever a call comes for its services in any part of the hospital.

